# Newborn Screening for Primary Immunodeficiency Diseases: History, Current and Future Practice

**DOI:** 10.1007/s10875-017-0455-x

**Published:** 2017-11-08

**Authors:** Jovanka R. King, Lennart Hammarström

**Affiliations:** 1 Department of Clinical Immunology, Karolinska University Hospital Huddinge, SE-141 86 Stockholm, Sweden; 2 Department of Immunopathology, SA Pathology, Women’s and Children’s Hospital Campus; Robinson Research Institute and Discipline of Paediatrics, School of Medicine, University of Adelaide, North Adelaide, South Australia 5006 Australia; 3 BGI-Shenzhen, Shenzhen, 518083 China

**Keywords:** Newborn screening, Primary immunodeficiency diseases, TREC, KREC, Next-generation sequencing

## Abstract

The primary objective of population-based newborn screening is the early identification of asymptomatic infants with a range of severe diseases, for which effective treatment is available and where early diagnosis and intervention prevent serious sequelae. Primary immunodeficiency diseases (PID) are a heterogeneous group of inborn errors of immunity. Severe combined immunodeficiency (SCID) is one form of PID which is uniformly fatal without early, definitive therapy, and outcomes are significantly improved if infants are diagnosed and treated within the first few months of life. Screening for SCID using T cell receptor excision circle (TREC) analysis has been introduced in many countries worldwide. The utility of additional screening with kappa recombining excision circles (KREC) has also been described, enabling identification of infants with severe forms of PID manifested by T and B cell lymphopenia. Here, we review the early origins of newborn screening and the evolution of screening methodologies. We discuss current strategies employed in newborn screening programs for PID, including TREC and TREC/KREC-based screening, and consider the potential future role of protein-based assays, targeted sequencing, and next generation sequencing (NGS) technologies, including whole genome sequencing (WGS).

## Introduction

Population-based newborn screening enables the early identification of asymptomatic infants with a range of severe diseases, for which effective treatment is available and where early diagnosis and intervention prevent serious sequelae. Primary immunodeficiency diseases (PID) are a heterogeneous group of inborn errors of immunity, the majority of which present in infancy and result in significant morbidity and mortality. Until recently, it was not possible to identify infants with PID prior to the onset of clinical symptoms, at which point they typically have complications of severe and protracted infection. Advances in technology have enabled identification of infants with severe forms of PID manifested by T and/or B cell lymphopenia. Here, we review the early origins of newborn screening and the evolution of screening methodologies. We review current strategies employed in newborn screening programs for PID, including T cell receptor excision circle (TREC) and kappa recombining excision circles (KREC)-based screening approaches, and discuss the potential future role of protein-based assays, targeted sequencing, and next generation sequencing (NGS) technologies.

### The Early Origins of Newborn Screening

#### Phenylketonuria: Discovery, Treatment, and Screening

Phenylketonuria (PKU), also known as Følling’s disease, was first identified in 1934 by the Norwegian biochemist and physician Ivar Asbjörn Følling (1888–1973), who evaluated two siblings aged 4 and 7 years with developmental delay and an unusual urinary odor [1]. He postulated that these features might be linked and set about identifying a potential causative compound in their urine. As a result of a series of experiments, he hypothesized that defective phenylalanine metabolism was responsible for their symptoms. A survey of 430 patients in a nursing home and school for children with intellectual disability led to identification of eight further cases, including two sibling pairs, with further pedigree analyses suggesting an autosomal recessive inheritance pattern [1, 2]. Følling named the disease “imbecillitas phenylpyruvica,” which in later years became known as “phenylketonuria,” a term coined by English geneticist Lionel Penrose [2]. The biochemical defect was described by George Jervis in 1945 [3]. In 1953, Bickel et al. highlighted the beneficial effects of a phenylalanine-free diet in a child with PKU whose developmental progress markedly improved, but regressed upon cessation of the dietary restriction [4]. In 1956, Horner and Streamer reported marked improvement (but not complete reversal) of behavior and development in two children with PKU aged 4 and 4.5 years treated with a low-phenylalanine diet [5]. In comparison, when the diet was commenced at 8 weeks of age in the younger sibling of one of these children, the child developed normally [5], providing evidence for the necessity of early institution of the low-phenylalanine diet in infants with PKU.

Although infants with affected siblings could be identified and treated early, it became clear that all infants should be tested for PKU in the newborn period to prevent long-term sequelae [2]. In the late 1950s, Willard Centerwall developed a “diaper test” to screen newborns for PKU using a ferric chloride reaction on freshly wet diapers, which enabled early identification and treatment of many infants with PKU in California [6]. However, one limitation was the absence of the compound in the urine until the child was a few weeks old [2], prompting development of a blood testing strategy which could be carried out in the first few days of life. Robert Guthrie (1916–1995), an American microbiologist, described a method of specimen collection from newborns, where blood from a heel puncture was applied to filter paper, dried, and then subjected to testing [7]. This later became known as the “Guthrie card,” and this method continues to be used in newborn screening programs worldwide. In 1963, Guthrie and Ada Susi described a method for detection of PKU in newborns based on the inhibition of growth of *Bacillus subtilis* by phenylalanine and associated compounds [7]. Small, circular punches taken from the dried blood spots (DBS) were placed on agar medium, and high levels of phenylalanine in PKU patients resulted in bacterial growth inhibition [7]. This marked the beginning of large-scale population screening in the USA, with the PKU screening program starting in Massachusetts in 1963, followed by rapid uptake in other states and in other countries around the world [8].

#### Recognition of the Important Role of Screening in Population Health

In 1968, James Maxwell Glover Wilson (1913–2006), Principal Medical Officer at the Ministry of Health, in London, England, and Gunnar Jungner (1914–1982), Chief of Clinical Chemistry at Sahlgren’s Hospital in Gothenburg, Sweden, published their World Health Organization (WHO) report entitled “Principles and practice of screening for disease” [9]. This was, and remains to this day, a highly significant contribution to the public health and population screening literature. Their description of the principles underpinning screening practices and the “Wilson and Jungner Criteria” for disease inclusion in screening programs (Table 1) [9] are still highly relevant in guiding decision-making.

**Table 1:** Wilson and Jungner principles of early disease detection

1	The condition sought should be an important health problem.
2	There should be an accepted treatment for patients with recognized disease.
3	Facilities for diagnosis and treatment should be available.
4	There should be a recognizable latent or early symptomatic stage.
5	There should be a suitable test or examination.
6	The test should be acceptable to the population.
7	The natural history of the condition, including development from latent to declared disease, should be adequately understood.
8	There should be an agreed policy on whom to treat as patients.
9	The cost of case-finding (including diagnosis and treatment of patients diagnosed) should be economically balanced in relation to possible expenditure on medical care as a whole.
10	Case-finding should be a continuing process and not a “once and for all” project.

Wilson and Jungner 1968 [9]

#### Beyond PKU: Expansion of Newborn Screening Programs

Following the successful implementation of newborn screening for PKU in several locations worldwide, attention was then given to expanding programs to screen for other types of metabolic disease and other conditions. In Sweden, screening for PKU commenced in 1965, followed by the addition of galactosemia (1967), congenital hypothyroidism (1980), congenital adrenal hyperplasia (1986), and biotinidase deficiency (2002). In 2010, an additional 19 disorders were added using tandem mass spectrometry and currently, 24 diseases are included in the national screening program.

#### Newborn Screening Methodologies

Through the work of these pioneers throughout history, newborn screening practices have evolved and expanded to include a wider number of screened diseases. Technological advances have enabled development of new assays, with improved sensitivity, specificity and capacity for automation [10]. From the first generation of PKU screening using ferric chloride reactions in neonatal diapers to Guthrie and Susi’s bacterial inhibition assay, the next significant milestone in newborn screening methodology was the advent of tandem mass spectrometry (MS/MS). This technology enables identification of a compound in a biological sample based on the mass/charge ratio and provides an opportunity to screen for several compounds simultaneously, hence increasing the number of screened disorders [8]. This modality continues to be used in newborn screening programs around the world. Other techniques include spectrophotometry, fluorometry, and immunoassay [10].

Screening methodologies have subsequently expanded to include DNA-based testing strategies. A DNA-based screening program for glutaric academia type 1 was established in the Canadian provinces of Manitoba and north-western Ontario in 1998. Here, a high incidence of this disorder has been noted in a local indigenous population, attributable to a single homozygous mutation in glutaryl-CoA-dehydrogenase [11]. Targeted sequencing of this mutation enabled identification of several affected infants, facilitating early institution of treatment [11]. Targeted genetic testing has also been included in newborn screening algorithms for cystic fibrosis, where an elevated immunoreactive trypsinogen measurement is followed by screening for a panel of *CFTR* (cystic fibrosis transmembrane conductance regulator) mutations [12]. A targeted genetic testing strategy has also been described for screening newborns for familial hemophagocytic lymphohistiocytosis (FHLH) due to *UNC13D* inversion mutations [13].

#### Newborn Screening Programs Worldwide

Therrell et al. recently published a comprehensive report on the status of newborn screening worldwide, which reflects the great variability in screening practices in different regions [14]. Most programs are structured to screen for a number of core disorders, along with secondary target disorders (typically organic and amino acidemias and fatty acid oxidation disorders) that are differential diagnoses of the core disorders [8]. As well as laboratory-based testing performed on dried blood spot samples, in many countries, newborns are also screened for hearing loss and cardiac disease using bedside assessment techniques [14]. There is currently a capacity to screen for over 50 different conditions, and diseases which are included in newborn screening programs or potential future inclusions are listed in Table 2. Decisions regarding disease inclusion at the local level should be guided by knowledge of the natural history of the disease, availability of treatment modalities, ability to decrease morbidity and mortality through screening and results of cost-effectiveness analyses [14].

**Table 2:** Included conditions in newborn screening programs

Amino acid disorders
Phenylketonuria
Maple syrup urine disease
Homocystinuria
Citrullinemia type I
Argininsuccinic aciduria
Tyrosinemia I
Other secondary conditions (argininemia, citrullinemia type II, hypermethioninemia, benign hyperphenylalaninemia, biopterin defects, tyrosinemia type II and III)
Organic acid disorders
Methylmalonic acidemia (with or without homocystinuria)
Glutaric acidemia type I
Propionic acidemia
3-Methylcrotonyl-glycinuria
3-Hydroxy-3-methyl glutaric aciduria
Holocarboxylase synthase deficiency
β-Ketothiolase deficiency
Isovaleric acidemia
Other secondary conditions (malonic acidemia, isobutyrylglycinuria, 2-methylbutyrylglycinuria, 3-methylglutaconic acidurias, 2-methyl-3-hydroxybutyric acidurias)
Fatty acid β-oxidation disorders
Medium-chain acyl-CoA deficiency
Very long-chain acyl-CoA dehydrogenase deficiency
Long-chain 3-hydroxyacyl-CoA dehydrogenase deficiency
Trifunctional protein deficiency
Carnitine transport defect
Carnitine palmitoyltransferase I deficiency
Carnitine palmitoyltransferase II deficiency
Carnitine acylcarnitine translocase deficiency
Glutaric acidemia type II
Other secondary conditions (short-chain acyl-CoA dehydrogenase deficiency, medium-short-chain K-3-hydroxyacyl-CoA dehydrogenase deficiency, medium-chain ketoacyl-CoA thiolase deficiency, 2,4-dienoyl-CoA reductase deficiency)
Lysosomal storage disorders
Krabbe’s disease
Pompe’s disease
Fabry disease
Gaucher disease
Niemann pick disease
Mucopolysaccharidosis I
Mucopolysaccharidosis II
Other lysosomal disorders
Other
Congenital hypothyroidism
Human immunodeficiency virus (HIV)
Toxoplasmosis
Severe combined immunodeficiency (SCID)
X-linked adrenoleukodystrophy
Glucose-6-phosphate-dehydrogenase (G6PD) deficiency
Hemoglobinopathies
Sickle cell disease
Congenital adrenal hyperplasia
Classic galactosemia
Biotinidase deficiency
Cystic fibrosis
Duchenne muscular dystrophy
Galactokinase deficiency
Galactoepimerase deficiency
Point of care testing
Hearing loss
Congenital heart disease
Proposed future conditions
Fragile X syndrome
Ornithine transcarbamylase deficiency
Wilson disease
Guanidinoacetate methyltransferase deficiency

Villoria et al. [8], Clague and Thomas [10], Therrell et al. [14]

### Primary Immunodeficiency Diseases

Primary immunodeficiency diseases (PID) are a heterogeneous group of inborn errors of immunity, which were first recognized in the 1950s with the description by Ogden Bruton (1908–2003) of a young boy with recurrent infections and agammaglobulinemia [15]. Since this time, the broad clinical spectrum of PID has been recognized, and well over 300 different genetic mutations resulting in PID have been described to date [16]. Patients with PID typically present with a predisposition to infection, and delayed diagnosis results in significant complications and associated increased morbidity and mortality. PID present with a spectrum of clinical phenotypes and are caused by different pathophysiological mechanisms. They may be broadly classified as follows: immunodeficiencies affecting cellular and humoral immunity, combined immunodeficiencies with associated or syndromic features, predominant antibody deficiencies, immune dysregulatory diseases, congenital defects of phagocyte number or function, innate or intrinsic immune defects, auto-inflammatory disorders, complement deficiencies, or PID phenocopies [16].

Severe combined immunodeficiency (SCID) is one of the most severe forms of PID and is manifested by a lack of T cells. B and NK cells may be variably absent depending on the molecular defect. This condition is an immunological emergency and requires prompt diagnosis and management. SCID is uniformly fatal without treatment. This condition came to public attention in the 1970s, when David Vetter (1971–1984), known as the “bubble boy,” was diagnosed with SCID and immediately after birth, was placed in a protective sterile environment at the Houston Children’s Hospital in Texas. Unfortunately, a matched donor for hematopoietic stem cell transplantation (HSCT) was not available, and David lived in the “bubble” until his death at age 13 years from EBV-associated lymphoma (https://primaryimmune.org/). David’s case was an example of how early diagnosis and management of SCID augment clinical progress. Recent evidence suggests that patient outcomes are markedly improved if definitive therapy with HSCT is performed before the age of 3.5 months, prior to the onset of severe infections and other complications [17]. Realistically, this is only achievable by early identification of infants with SCID through newborn screening programs.

### History of Newborn Screening for Primary Immunodeficiency Diseases

#### Screening for Severe Combined Immunodeficiency (SCID)

Severe combined immunodeficiency (SCID) is an important health problem, for which the natural history is known and treatment is available, and as such, it meets the Wilson and Jungner criteria [9] and is a suitable candidate for population screening. SCID manifests with low or absent T lymphocytes, and thus screening newborns for T cell lymphopenia is an ideal strategy for identifying the disease. The first strategy proposed involved screening each newborn with a full blood count to determine the lymphocyte count [18], which was deemed to lack sensitivity. Subsequently, screening cord blood for T cell populations by flow cytometry was also considered [19]; however, given that this was likely to be time-consuming and expensive, other screening methods for the detection of T cell lymphopenia were considered.

T cell receptor excision circles (TREC) are small, circular pieces of episomal DNA which are formed during T cell receptor (TCR) rearrangement in naïve T cells and are thus surrogate markers for recent thymic emigrants. TREC were first visualized by electron microscopy as circular, extra-chromosomal DNA in mouse thymocytes in 1982 [20] and were later demonstrated to be the product of TCR rearrangement [21]. The TREC assay was developed by Douek et al. who demonstrated that TREC were specific to naïve T cells and described an age-related decline in healthy individuals and reduced levels in HIV infection [22]. TREC were noted to be stable, not prone to degradation, and did not replicate with subsequent cell division, making them an ideal marker for naïve T cell production. In 2005, Kee Chan and Jennifer Puck first described application of the TREC assay for large-scale population screening for SCID and other forms of T cell lymphopenia [23]. The TREC assay was subsequently optimized, and the first state-wide SCID screening pilot study was commenced in Wisconsin in 2008, led by Jack Routes and James Verbsky [24]. Later that year, the first child with SCID who was identified by newborn screening was successfully transplanted (Jeffrey Model Foundation, http://www.info4pi.org). Subsequently, screening was implemented in Massachusetts, Louisiana, and New York in 2009, and California, Texas, and Pennsylvania in 2010. Screening for SCID is now routinely conducted or due to commence in the near future in the majority of states, including the District of Columbia, Navajo Nation and Puerto Rico, with 92% of American infants undergoing screening at the present time [25, https://primaryimmune.org/idf-advocacy-center/idf-scid-newborn-screening-campaign/].

Although identification of infants with SCID was the intended aim of TREC-based newborn screening programs, it became evident that in addition to this disorder, the assay would also identify infants with T cell lymphopenia due to other primary and secondary causes (Table 3). For example, low TREC levels have been detected in individuals with 22q deletion syndrome, CHARGE association, and Trisomy 21 [26]. In addition, infants with forms of PID other than SCID may have low TREC, for example in ataxia telangiectasia and combined immunodeficiency diseases (CID). Thus far in prospective pilot studies, many cases of CID without an identifiable molecular cause have been detected using the TREC assay [27, 28, 30] and these patients require clinical characterization and long-term follow-up.

**Table 3:** Disorders detectable by TREC and KREC screening

Low TREC levels	Low KREC levels
Severe combined immunodeficiency* 22q deletion syndrome Combined immunodeficiency Ataxia telangiectasia DOCK 8 deficiency EDA-ID Trisomy 21 Trisomy 18 Kabuki syndrome CHARGE syndrome Noonan syndrome Jacobsen syndrome Nijmegen breakage syndrome** Fryns syndrome Schimke immuno-osseous dysplasia Cartilage hair hypoplasia CLOVES ECC Rac2 defect Renpenning syndrome TAR Other cytogenetic abnormalities - Including 6p deletion, ring chromosome 14, ring chromosome 17, chromosome 17p duplication, 14q microdeletion	Severe combined immunodeficiency (T-B-)** X-linked agammaglobulinemia (XLA) XLA-like disorders Nijmegen breakage syndrome**
Secondary causes	
Prematurity Congenital cardiac disease Chylothorax Multiple congenital anomalies Gastrointestinal anomalies - Including gastroschisis Third space losses Vascular leakage Hydrops Neonatal leukemia Maternal autoimmune disease Maternal HIV infection Maternal immunosuppression Other maternal medications -Including ritodrine	Maternal immunosuppression Other maternal medications - Including ritodrine

*DOCK8*, dedicator of cytokinesis 8; *CHARGE*, coloboma, heart defects, atresia choanae, growth retardation, genital abnormalities, ear abnormalities; *CLOVES*, congenital, lipomatous, overgrowth, vascular malformations, epidermal nevi, spinal/skeletal anomalies, and/or scoliosis; *ECC*, ectodermal dysplasia, ectrodactyly, and clefting; *TAR*, thrombocytopenia and absent radius; *HIV*, human immunodeficiency virus; *EDA-ID*, ectodermal dysplasia-associated immunodeficiency

*Excluding Zap70 deficiency, MHCII deficiency, and late-onset ADA deficiency

**Low TREC and KREC levels

Jyonuchi et al. [26], Kwan et al. [27], Chien et al. [28], Barbaro et al. [29]

In addition to TREC analysis, other SCID screening approaches have been proposed. In Italy, newborns are screened for SCID with the TREC assay, along with tandem mass spectrometry to identify ADA deficiency, which also enables identification of infants with delayed-onset ADA deficiency [31–33]. A similar approach has been described to identify patients with PNP deficiency [34]. A two-tiered testing approach using TREC analysis combined with IL-7 measurement has also been proposed as a means to increase the specificity of SCID screening [35]; however, this has not yet been optimized for application in large-scale screening programs [36].

Primary immunodeficiency diseases were previously thought to be rare entities, and the incidence of SCID was unknown. Prospective screening programs have since enabled the true incidence of SCID to be determined. Furthermore, screening has facilitated the identification and treatment of infants who would have otherwise died from complications of undiagnosed SCID. Based on studies in the USA where over 3 million infants were screened, the incidence of SCID was found to be much higher than expected at 1/58000 and the incidence of clinically relevant T cell lymphopenia was 1/7300 [27]. The annual live birth rate in the USA is approximately 4 million. Given that it was previously estimated that the incidence of SCID was 1/100000, screening has thereby increased the number of expected cases from approximately 40 to 69 annually, equating to an additional 29 cases each year identified and managed with potentially curative treatment. Given that PID constitute a large and heterogeneous group of genetic disorders which are individually rare entities, newborn screening offers additional benefits beyond individual patient care. Screening provides important opportunities to increase our knowledge of the clinical and pathophysiological spectrum of PID and gain additional experience in management of these conditions, particularly as affected infants are identified early in their disease course. Newborn screening thereby facilitates continued, collaborative research and progression of our knowledge in this field by enabling collation of rare and diverse cases and building national and international expertise.

#### Screening for Congenital B Cell Deficiency Disorders

Mutations in key genes which are essential for B cell ontogeny give rise to congenital B cell deficiency disorders including X-linked agammaglobulinemia (XLA) (resulting from a mutation in the *BTK* gene) and autosomal recessive XLA-like disorders. Patients have absent B cells, extremely low or undetectable immunoglobulin levels, and an increased susceptibility to severe infection with bacteria and other pathogens [16]. Like T cells, B cells also undergo rearrangement of the variable, diversity, and joining domains (V(D)J recombination) during development in order to produce unique B cell antigen receptors, and this process also yields episomal, circular DNA referred to as a kappa recombining excision circles (KREC). V(D)J recombination within the *IGK* locus results in a Vk-Jk coding joint, followed by rearrangement of the intron RSS and Kde elements with deletion of the Ck exon and enhancers. The coding joint remains present in the genome, whereas the KREC with the corresponding signal joint is excluded as a stable, circularized DNA fragment [37]. In 2007, van Zelm et al. described this process and developed a KREC assay using a PCR-based method [37]. They demonstrated that KREC levels reflected the replication history of B cells and had potential utility in assessing B cell recovery following HSCT and in the assessment of patients with antibody deficiency disorders such as common variable immunodeficiency (CVID) [37]. In 2011, Nakagawa et al. were the first to demonstrate the utility of the KREC assay in identifying newborns with B cell deficiency disorders, showing that signal joint KREC were absent in blood and Guthrie card specimens in patients with XLA [38].

#### Concurrent Screening for Severe Forms of Primary Immunodeficiency Manifested by T and B Cell Lymphopenia

Given the potential utility of the KREC assay in newborn screening for PID, a multiplexed TREC/KREC assay has been described, which enables simultaneous identification of infants with severe forms of PID manifested by T and/or B cell lymphopenia (Table 3 and 4) [40]. This approach has since been adopted in newborn screening pilot studies in Sweden and Spain [29, 41]. Multiplexed TREC/KREC assays offer many advantages over TREC screening alone, as well as identification of congenital B cell defects; it enables identification of individuals with different forms of PID which might be missed by a TREC-alone assay, including late-onset ADA deficiency, some cases of Nijmegen breakage syndrome and other selected disorders [40].

**Table 4:** Results of prospective newborn screening programs for primary immunodeficiency

Region	Screening period	Screening strategy	Number of newborns screened	Primary immunodeficiency cases identified	References
USA (10 states + Navajo region)	January 2008–July 2013 (5.5 years)	TREC	3,030,083	SCID (*n* = 52) - Typical SCID (*n* = 42) - *IL2RG* (*n* = 9) - *IL7RA* (*n* = 6) - *ADA* (*n* = 5) - *RAG1* (*n* = 4) - *JAK3* (*n* = 3) - *DCLRE1C* (*n* = 1) - *RAG2* (*n* = 1) - *CD3D* (*n* = 1) - *TC7A* (*n* = 1) - Pallister-Killian syndrome; tetrasomy 12p (*n *= 1) - Molecular defect unknown (*n* = 6) - Genetic testing incomplete (*n* = 4) - Leaky SCID (*n* = 10) - *RAG1* (*n* = 4) - *RMRP* (*n* = 2) - *IL2RG* (*n* = 1) - *DCLRE1C* (*n* = 1) - Molecular defect unknown (*n* = 2)	Kwan et al. 2014 [27]
Taiwan	2010–2017 (78 months)	TREC	920,398	SCID (*n* = 7) - *IL2RG* (*n* = 3) - *RAG1* (*n* = 1) - Molecular defect unknown (*n* = 3) SCID variant, molecular defect unknown (*n* = 8) EDA-HT (*n* = 1)	Chien et al. 2017 [28]
Sweden (Stockholm county)	15 November 2013–14 November (3 years)	TREC/KREC	89,462	SCID (*n* = 2) - Artemis deficiency) (*n* = 1) - *ADA* deficiency (*n* = 1) Ataxia telangiectasia (*n* = 1) CID, molecular defect unknown (*n* = 2)	Barbaro et al. 2016 [29] Zetterström et al. 2017 [30]
Israel	1 October 2015–30 April 2017 (18 months)	TREC	290,864	SCID (*n* = 13) - Typical SCID (*n* = 10) - *DCLRE1C* (*n* = 3) - *IL7RA* (*n* = 2) - *RMRP* (*n* = 1) - Ligase 4 deficiency (*n* = 1) - Complete DiGeorge Syndrome (*n* = 1) - Molecular defect unknown (*n* = 2) - Leaky SCID (*n* = 3) - *DCLRE1C* (*n* = 2) - MHC2 deficiency*/RFX5* (*n* = 1) Undefined PID (*n* = 6)	Rechavi et al. 2017 [39] Rechavi et al. (personal communation)

*TREC* T cell receptor excision circles, *KREC* kappa recombining excision circles, *SCID* severe combined immunodeficiency, *CID* combined immunodeficiency, *EDA-HT* X-linked recessive anhidrotic ectodermal dysplasia-associated immunodeficiency

#### Limitations of Current Screening Strategies for Primary Immunodeficiency

It has been established that although TREC screening will identify the majority of infants with SCID, cases where the molecular defect lies downstream of T cell receptor rearrangement will not be detected. This includes Zap70 deficiency, MHC Class II deficiency, and some cases of delayed ADA deficiency [42–47]. Defects of T cell function despite a quantitatively normal T cell number will also not be detected by the TREC assay.

### The Current Status of Newborn Screening for Primary Immunodeficiency Diseases Worldwide

Screening programs for PID have been instituted in many regions. TREC-based PID screening programs have been established in the majority of American states (including the Navajo region, District of Columbia, and Puerto Rico), Taiwan, Israel, Qatar, and several Canadian regions, and are due to commence in New Zealand in the near future [48]. Screening programs utilizing TREC-only, TREC/KREC, or TREC/ADA strategies have also been evaluated in pilot studies in Italy, Sweden, Germany, The Netherlands, Japan, France, Spain, Norway, France, the UK, Turkey, Slovenia, Saudi Arabia, Iran, Iceland, Denmark, and Brazil, and many more regions have applications in progress to commence screening programs [48].

### The Future of Newborn Screening for Primary Immunodeficiency Diseases

#### Screening for Complement and Granulocyte Disorders Using Protein-Based Methods

Complement proteins are an important component of the innate immune system, and deficiencies in complement give rise to a variable clinical phenotype, including autoimmune disease, renal disease, and a susceptibility to specific bacterial infections which frequently result in life-threatening infection [16]. Disorders of granulocyte number and function are an important group of disorders, with affected individuals typically presenting with severe bacterial or fungal infections and other features such as colitis [16]. Atypical presentations frequently result in delayed diagnosis and treatment. Patients with complement or granulocyte disorders will not be identified using current TREC or TREC/KREC-based methodologies, which identify PID manifesting with T and/or B cell lymphopenia, respectively. As such, protein-based screening methodologies have been proposed as a means by which to identify infants with complement and granulocyte disorders. Specific complement proteins, including C2 and C3, can be eluted from DBS and quantified, enabling identification of infants with low or undetectable protein levels at birth [49, 50], facilitating early intervention with prophylactic measures to prevent potentially catastrophic outcomes. It has also recently been demonstrated that complement deficiency can be identified using a whole genome sequencing (WGS)-based newborn screening strategy [51].

#### Screening for Familial Hemophagocytic Lymphohistiocytosis

Targeted DNA sequencing has previously been employed as a screening strategy for selected diseases, such as glutaric acidemia type I and cystic fibrosis. This approach has also been described as a potential method by which infants with familial hemophagocytic lymphohistiocytosis (FHLH) due to mutations in *UNC13D* may be identified [13]. Fifty percent of FHLH cases in Scandinavia are due to homozygous *UNC13D* inversion mutations, and it has been demonstrated that a reduction in the wild type gene copy numbers is an effective way to screen for affected individuals [13].

#### The Role of Next Generation Sequencing in Newborn Screening for PID

Primary immunodeficiency diseases are a heterogeneous group of disorders, which differ in terms of clinical phenotype, laboratory findings, and underlying molecular abnormalities. There are currently over 300 different genetic mutations associated with PID [16], and this number continues to increase. As such, there is no single test which can reliably identify all infants with PID at birth. Rapid advances in genomic medicine have resulted in increased availability and reduced costs of next-generation sequencing (NGS), and whole exome sequencing (WES) and whole genome sequencing (WGS) have an established role in diagnostic medicine. Previously, the time taken from sample collection to receipt of results of NGS-based studies has been prolonged, often taking weeks or months. However, “rapid” NGS has been described in the setting of critically ill infants in the pediatric intensive care unit (PICU) and neonatal intensive care unit (NICU) setting, where a 26 hour turnaround time has been achieved, and results have impacted upon patient care [52–54]. In one cohort of 35 acutely unwell infants, 20 were diagnosed with a genetic disease using rapid WGS, 13 of whom had de novo mutations identified, and the diagnosis directly influenced management decisions (including specific management or palliation) in 10 cases [52]. It follows then, that in addition to diagnostic medicine, NGS is likely to have a future role in newborn screening for PID and other conditions.

The National Institutes of Health is currently evaluating the role of up-front NGS in newborn screening as part of the NSIGHT (Newborn Sequencing in Genomic Medicine and Public Health) project [55]. Whole-exome-based newborn screening for currently screened and additional disorders will be evaluated, along with the experience of parents and clinicians in the exchange and utility of genomic information. The ethical, legal, and social implications will also be explored [55]. In addition, rapid NGS in the NICU setting will be further evaluated [55].

Recently, Pavey et al. used WGS to screen 1349 newborn and parent trios for variants in 329 known PID-associated genes [51]. Applying a genotype-first pipeline, pathogenic or likely pathogenic mutations were identified in 396 infants; however, only one was found to have a genomically predicted PID (complement component C9 deficiency). A phenotype-first approach resulted in identification of 29 infants in the cohort who were potentially immunodeficient based on clinical features; however, no mutations were identified in the interrogated PID genes. Pathogenic mutations were identified in other (non-PID associated) genes in three of the children [51]. Lucarelli et al. also recently described a WGS-based screening strategy for cystic fibrosis, involving interrogation of a panel of 188 *CFTR* mutations [56]. Bodian et al. also evaluated up-front WGS as a screening strategy to evaluate 1696 newborns for variants in 163 genes which are implicated in diseases which are currently screened for in the USA, demonstrating that WGS was complementary to conventional newborn screening and gave fewer false positive results, resolved inconclusive findings and provided more precise diagnostic information compared to conventional techniques [57].

Applying a WGS-based approach to newborn screening represents a change in paradigm, and there are many factors which must be taken into consideration before adopting this approach in population-based screening programs [48, 58]. Firstly, candidate diseases and target genes for evaluation should be identified. In the case of PID, an ideal starting point would be establishing a panel of the currently identified 300+ PID-associated genes, which would need to be updated as new genes are described. Care must be taken to screen only for diseases with an established genotype-phenotype correlation. Robust and cost-effective testing systems must be established. The tests must be appropriately sensitive and specific, with an agreeable turnaround time and data should be analyzed and managed appropriately. An appropriate pipeline should be established to manage abnormal results, including confirmatory and second-tier testing and seamless integration with clinical services. The cost-effectiveness of this screening approach must also be formally assessed. Ethical, legal, and social implications are major considerations, including issues pertaining to consent, biobanking of genetic material, and data and implications of genetic findings for other family members. A plan must also be in place for management of variants of unknown significance, including mutations in genes known to cause debilitating or lifespan-reducing disease for which there is no known treatment [48]. A screening approach using up-front WGS must be evaluated in large, prospective trials prior to adopting this strategy in population-based newborn screening programs. We support the consideration of WGS as an up-front screening method.

Management of PID is variable and is dependent on the disease, clinical phenotype, and molecular defect. In the case of SCID, HSCT or gene therapy is currently available curative therapies. Advances in CRISPR-Cas based genome editing have led to interest in potential therapeutic applications of this modality. Scott and Zhang recently highlighted that prior to institution of CRISPR-based therapy, patients should be screened using WGS to ensure safety and reduction of off-target effects [59]. The early identification of a specific PID-associated mutation as a result of newborn screening by NGS will enable timely initiation of the aforementioned therapies, along with other targeted, personalized treatment options [60].

#### Prenatal Screening

Newborn screening enables identification of infants with key disorders requiring early intervention soon after birth. Prenatal screening for disorders such as Trisomy 21 and neural tube defects is well established in obstetric practice, along with prenatal diagnostics in the case of a high risk of a specific genetic disorder based upon positive family history or other factors. However, this has typically required invasive procedures which carry associated risks, such as amniocentesis and chorionic villus sampling. Technological advances have resulted in the ability to detect fetal DNA in maternal plasma, and Lo et al. demonstrated that massively parallel sequencing (MPS) enables detection of fetal aneuploidies such as trisomies 21, 13, and 18 [61]. In addition to chromosomal aneuploidy, this technique also enables detection of sub-chromosomal deletions and duplications including 22q11 (DiGeorge Syndrome) and 5p (Cri-du-chat Syndrome) deletions and deletions associated with Prader-Willi and Angelman Syndromes. Monogenic traits and disorders such as RhD status and *FGFR3* mutations which give rise to achondroplasia can also be identified. Targeted or whole genome sequencing of the fetus is also possible, along with sequencing of the fetal transcriptome and methylome, enabling identification of a wide range of disorders [62]. Prenatal screening technologies may well also become part of the changing landscape of screening practices for PID and other disorders in the future.

## Conclusion

Newborn screening practices worldwide have evolved significantly since the discovery of PKU in 1934, with rapid development of new and improved assays to enable identification of a wider range of conditions in infants and facilitating early diagnosis and treatment, and improving clinical outcomes. Primary immunodeficiency diseases are a heterogeneous group of disorders, and no single assay at the present time will identify all forms of PID, necessitating a challenge of current newborn screening paradigms. In the genomic era, it is likely that this will involve up-front next generation sequencing, including whole exome sequencing and ultimately, whole genome sequencing. This approach must be evaluated in large, prospective trials prior to adopting this strategy in population-based newborn screening programs.
